# Continuous glucose monitoring in kidney transplant recipients: a narrative review

**DOI:** 10.1186/s12882-025-04691-2

**Published:** 2025-12-18

**Authors:** Khaled Oweidat, Benjamin C. T. Field, Christopher K. Farmer

**Affiliations:** 1 Department of Renal Medicine, East Kent Hospitals University NHS Foundation Trust, Canterbury, Kent UK; 2 Kent and Medway Medical School, University of Kent and Canterbury Christ Church University, Canterbury, Kent UK; 3 Section of Clinical Medicine, School of Biosciences, Faculty of Health and Medical Sciences, University of Surrey, Guildford, Surrey UK; 4 Department of Diabetes and Endocrinology, Surrey and Sussex Healthcare NHS Trust, Redhill, Surrey UK; 5 Centre for Health Services Studies, University of Kent, Canterbury, Kent UK

**Keywords:** Continuous glucose monitoring, CGM, Kidney transplantation, Renal transplantation, Post-transplant diabetes mellitus, PTDM, New-onset diabetes after transplant, NODAT, Glycemic variability, Graft survival

## Abstract

Continuous glucose monitoring (CGM) has transformed diabetes management, offering real-time and dynamic insights into glucose variability and addressing the limitations of traditional glucose assessment methods. Kidney transplantation, the most common solid organ transplant, carries a considerable burden of post-transplant diabetes mellitus (PTDM), which is linked to increased cardiovascular events, graft dysfunction, and increased mortality. This review explores the role of CGM in kidney transplant recipients, particularly its impact on glycemic profiles and its predictive value for post-transplant diabetes mellitus (PTDM). At the time of this review, CGM had not yet been incorporated into standard transplant care protocols. Evidence shows that perioperative CGM outperforms traditional tests in identifying frequent hyperglycemia and glycemic variability in the first weeks after transplantation, enabling enhanced glycemic control and improving the recipient’s clinical outcome. Studies demonstrate higher glucose variability in kidney only recipients compared to other organ recipients, and in type 2 diabetes patients compared to those with PTDM. Poor perioperative glycemic control and glycemic variability detected by CGM have been linked to acute rejection and reduced graft survival. CGM-derived metrics outperform conventional glucose measures in predicting PTDM. CGM metric thresholds within the first month post-transplant achieved sensitivities above 85% and specificities up to 83% for PTDM risk. CGM-guided adjustment of immunosuppressants and steroid dosing have been shown to reduce hyperglycemia and variability. Comparative studies indicate that glycosylated hemoglobin A1c correlates poorly with CGM in the early post-transplant period, often misclassifying patients as normoglycemic. CGM appears to offer clinically relevant insights for the early detection, prediction, and management of dysglycemia in kidney transplant recipients.

## Background

Continuous glucose monitoring (CGM) has become a transformative technology in diabetes management. Compared with traditional monitoring methods, which rely on intermittent blood or urine sampling only offering a fragmented view of glucose dynamics, CGM continuously tracks glucose levels in the interstitial fluid, delivering a wealth of information in real time on the variability of glycemia [1].

The European Association for the Study of Diabetes (EASD), in collaboration with the American Diabetes Association (ADA), advises CGM for all individuals with type 1 diabetes and for those with type 2 diabetes who are using multiple daily insulin injections or insulin pump therapy [2]. The use of CGM has been identified as a beneficial tool for individuals with type 2 diabetes who are not using insulin to achieve certain glycemic goals. Its use is particularly warranted in those experiencing recurrent or severe hypoglycemia, as well as those with impaired hypoglycemia awareness [3].

Kidney transplantation remains the most effective treatment for eligible patients with end-stage renal disease (ESRD). In 2023, the Eurotransplant region, which includes eight European countries, recorded a total of 3,161 deceased-donor kidney transplants. The number of active patients on the kidney transplant waiting list within the region has reached 13,498 [4]. Kidney transplantation continues to be the most commonly performed solid organ transplant in Europe and globally, with a median of approximately 27–35 kidney transplants per million population per year in Europe [5]. Worldwide, there was a median of 14 kidney transplantations per million population and a prevalence of 255 per million population in 2018 [6].

The prevalence of diabetes and prediabetes prior to kidney transplantation is notably high, with 17% to 32% of transplant recipients having diabetes at the time of transplantation [7, 8]. Additionally, up to 55% of candidates on the kidney waiting lists are either diabetic or prediabetic [9].

Post-transplantation diabetes mellitus (PTDM) refers to both new onset of diabetes mellitus post-solid organ transplant (NODAT) and previously undiagnosed pre-existing diabetes [10]. The incidence of post-transplant diabetes mellitus (PTDM) in the first year following transplantation (PTDM) ranges from 10% to 74%, depending on various factors such as demographics and immunosuppressive regimens [11, 12]. Compared with other solid organ recipients, kidney transplant recipients have been found to have the highest PTDM incidence [13].

A cohort study showed that 13.8% of the candidates with kidney failure on the transplantation lists between 2000 and 2019 had diabetes as the primary cause of kidney disease. In addition, the study highlighted that people with diabetes who have been listed for a kidney transplant are at significantly higher risk of not progressing to transplantation compared to those with other kidney failure-related conditions [14].

PTDM has consequences for transplant recipients. It is associated with increased risks of cardiovascular complications, graft dysfunction, and mortality [10, 15, 16]. Poor glycemic control in the early post-transplant period has been found to exacerbate ischemia-reperfusion injury, potentiate alloimmunity, and exaggerate the inflammatory responses. These mechanisms have been linked to graft rejection and the modulation of the immunosuppressive process [16, 17]. PTDM is also associated with an increased likelihood of returning to dialysis and affects long-term graft survival, although episodes of acute rejection requiring high-dose corticosteroid therapy may act as important confounders in the association between PTDM and graft dysfunction [15].

Glycemic management in kidney transplantation has been viewed as an issue of concern, with studies reporting that hyperglycemia occurs in more than 70% of diabetic patients consistently within the first three years after kidney transplantation [7]. Transplant recipients with pre-existing diabetes tend to have significantly higher hemoglobin A1c (Hb1Ac) levels compared with those without diabetes and those who develop PTDM [7, 15]. Factors associated with poor glycemic control after transplantation include receiving a graft from a deceased donor, age, and dialysis modality prior to transplantation [7].

Despite the growing recognition of PTDM as a significant complication in kidney transplant recipients, the commonly used methods for glycemic assessment - HbA1c, fasting plasma glucose (FPG), and oral glucose tolerance tests (OGTT) - have been reported to have limitations, particularly during the early post-transplant period. These limitations may delay diagnosis and hinder timely intervention [18, 19].

Continuous glucose monitoring (CGM) has emerged as a promising technology capable of overcoming some of the limitations of the traditional methods. The ability to capture real-time glucose dynamics and glycemic variability may allow earlier detection of dysglycemia, more accurate risk stratification for PTDM, and improved tailoring of immunosuppressive and antidiabetic therapy, hence improving graft outcome. Despite the potential advantages, continuous glucose monitoring (CGM) is not currently standard practice or is extensively utilized in kidney transplant care protocols.

This review aims to explore current evidence on the potential benefits of CGM in kidney transplant recipients. The study explored the glycemic profile changes that occur around kidney transplantation and assessed the existing evidence around the role of CGM as a predictive tool for the risk of developing post-transplant diabetes mellitus (PTDM). By synthesizing the available research, this review aims to provide insights into the utility of CGM in kidney transplant recipients and to highlight opportunities for future research and clinical implementation.

## Identification of articles

We conducted a comprehensive search of the EMBASE, MIDLINE, Google Scholar, and PubMed databases for relevant articles. The search terms included Continuous Glucose Monitoring, CGM, Ambulatory Glucose Monitoring, Real-Time Continuous Glucose Monitoring, Flash Glucose Monitoring, Freestyle Libre, Dexcom, Intermittent Continuous Glucose Monitoring, rtCGM, isCGM, Ambulatory Glucose Profile, Kidney Transplant, Renal Transplant, Kidney Transplantation, Renal Allograft, Kidney Allograft, Renal Graft, and Kidney Graft. Our search covered articles published in English up to October 2024.

This report is a narrative review that includes clinical studies, observational research, case reports, and conference abstracts. Articles that did not involve CGM use and were not for the kidney transplant population were excluded.

The review was narrative in nature; no formal quality scoring or bias assessment was applied. Relevant articles from the search were screened by two reviewers, and the included articles were then classified under the four themes and subtopics that formed the body of our review.

## Glycemic variability is a potential risk in kidney transplant recipients: insights highlighted by continuous glucose monitoring in the perioperative period

Fluctuations in blood glucose throughout the day, known as glycemic variability (GV), have been identified as independent risk factors for the development of microvascular and kidney complications in patients with diabetes mellitus [20–22]. Evidence suggests that these fluctuations, rather than high glucose alone, may contribute to endothelial dysfunction and oxidative stress, both of which are associated with microvascular diabetic complications [22, 23]. CGM has emerged as a key tool for assessing glycemic variability by allowing minimally invasive glucose monitoring within and outside healthcare and research settings [24].

The use of CGM in the post-transplant period revealed that after kidney transplantation, hyperglycemia (≥ 11.1 mmol/L) occurred in 79% of patients in the first days following kidney transplantation [25]. Rodríguez et al. [26] highlighted the benefits of CGM over older monitoring methods for assessing glucose profiles and identifying hyperglycemia in transplant recipients. Their study used CGM to compare glucose variability between those who underwent simultaneous pancreas-kidney (SPK) transplantation and those who received kidney-alone transplants, demonstrating that SPK recipients experienced significantly fewer hyperglycemic excursions than kidney-alone recipients.

In a related study, CGM was used to explore the nature of glycemic variability among non-transplanted individuals with type 2 diabetes mellitus (T2DM) and those with PTDM [27]. The study revealed that glycemic variability was significantly higher in participants with T2DM than in PTDM recipients, highlighting potential pathophysiological differences between the two conditions. The study highlights the significance of CGM as a tool for understanding the mechanism of diabetes development in transplant recipients. Similar findings were reported by Aouad et al. [25], who reported that recipients with preexisting diabetes exhibited higher glucose variability than those who developed NODAT. A significant increase in glycemic variability was observed within the first two weeks after kidney transplant surgery compared with preoperative levels, the rise was not reflected in fasting glucose measurements. The study included both non-diabetic and diabetic participants [28].

Reports of the short-term impact of diabetes mellitus, particularly poor glycemic control status post-transplant and PTDM, have demonstrated a significant increase in major cardiovascular events in kidney transplant recipients, return to dialysis, and death within one year after kidney transplantation compared to non-diabetic recipients [15]. A multicenter study found that high Hb1Ac levels were a significant predictive factor of long-term graft survival in diabetic patients and were associated with poorer long-term graft outcomes. Evidence further indicates that strict pre-transplant glycemic control is an important determinant of successful graft outcomes [16]. Poor glycemic control during the perioperative kidney transplantation period in diabetic patients is associated with an increased risk for acute graft rejection, as hyperglycemia potentially exacerbates ischemic reperfusion injury in the early post-transplant period [16, 17]. It also contributes to chronic graft rejection and reduced graft survival by partly amplifying the toxic effect of immunosuppressant medication [17]. Table 1 summarizes the key studies that have utilized CGM to assess glycemic variability in the perioperative period following kidney transplantation.

**Table 1:** Summary of studies in which CGM was used to assess glycemic variability during the kidney transplant perioperative period

Study	Year	Population	CGM Period	Main CGM Metric(s)	Key Findings
Aouad et al.	2018	28 kidney transplant recipients (3 of them had diabetes prior to transplantation)	3–5 days at three different time points (day of transplant, month 3, and month 6 post-transplant	Glucose variability, Mean glucose, MAGE*, % time with hyperglycemia	-79% had post-op hyperglycemia in the first 2 months post-transplant. -Diabetes control was inferior in participant who developed NODAT -Glucose variability was significantly increased in prior diabetic recipients.
Rodríguez et al.	2010	SPK** vs. KT*** recipients ( 6 SPK and 2 KT type 1 diabetic recipients)	Early post- operation (48 h post operation)	Mean glucose, glucose excursions, GV^††^	SPK patients had fewer excursions; CGM was better than traditional methods
Werzowa et al.	2015	18 recipients (10 with T2DM vs. 8 PTDM vs. 10 non-transplant T2DM subjects	Minimum of 6 months post-transplant	-Variety of values, including GRADE^†^, - Variety of glycemic control indexes	T2DM patients showed greater GV than PTDM, both PTDM glycemic control worse than normal non transplant diabetic control
Jo et al.	2022	72 KT recipients not known if they were diabetic	Perioperative (2 weeks pre and 2 weeks post-transplant)	Pre- vs. post-op GV & GMI ^†††^	Significant GV increase in post-transplant

*Mean amplitude of glycemic excursions

** Simultaneous Pancreatic – Kidney Transplantation

*** Kidney Transplantation

^†^ Glycemic Risk Assessment Diabetes Equation

^††^ GV: Glucose variability

^†††^ Glucose Management Indicator

The current evidence, while it represents the first attempt to understand the glycemic profile around the perioperative area, lacks clinical significance. The available study populations are small. Early work by Aouad et al. represents one of the first applications of CGM in the transplant population but included a small cohort with intermittent monitoring for a small sample size. Similarly, the study by Rodríguez et al. involved only eight patients and had a short monitoring duration. While these studies underscore CGM’s potential as a powerful exploratory tool, their conclusions regarding glycemic pattern differences between SPK and kidney-only recipients remain subject to confounding.

None of the studies reviewed in this section have a design that effectively assesses the impact of glycemic variability on graft outcomes on the basis of CGM metrics. All the studies were observational and lacked designs that accounted for confounders and related glucose variability. Larger, rigorously designed studies are needed with a focus on the clinical implications of CGM findings for patient outcomes.

## Predictive value of CGM for post-transplant diabetes mellitus (PTDM)

Given that PTDM is associated with accelerated graft failure and cardiovascular complications, it has been hypothesized that prevention or early detection and treatment of PTDM might improve graft survival and decrease mortality in the transplant population [10, 29].

The risk factors for PTDM overlap with conventional diabetes mellitus risk factors such as age, BMI, ethnicity, and transplant-related risk factors such as immunosuppressant type and regimen, cytomegalovirus infection, and hepatitis C virus infection [30, 31]. The use of calcineurin inhibitors (particularly tacrolimus) and glucocorticoids, which are both known to increase the incidence and severity of hyperglycemia. Their minimization is key to reducing the incidence of PTDM; however, this is challenging as they are often required to prevent transplant rejection.

The recommended post-kidney transplantation maintenance therapy includes calcineurin inhibitors (preferably tacrolimus) and an anti-proliferative agent, likely mycophenolate, with or without the addition of steroids. By 2–4 months post-transplant, maintaining the lowest dose of immunosuppressant medication while continuing calcineurin inhibitors is highly recommended [32].

The considerable improvement in kidney transplant outcomes since the 1980s is largely due to lower acute rejection, which is attributed to advances in immunosuppressive medication. New strategies aimed at sparing or minimizing the use of calcineurin inhibitors and steroids have emerged to reduce the long-term toxic effects of immunosuppressive medication in kidney transplantation. Steroid-sparing in well-designed trials has significantly increased acute graft rejection [33]. However, steroid-free protocols have shown that interstitial fibrosis and tubular atrophy are more common in steroid-free recipients, potentially compromising long-term graft survival [33]. Moderate to high-strength evidence has shown that calcineurin inhibitor withdrawal can be performed without impacting the risk of long-term survival, although it increases the risk of acute rejection [33, 34]. Switching to another agent, such as an inhibitor of the mammalian target of rapamycin (mTOR), has shown an improvement in kidney function and decreased graft injury, although with a trade-off of increased side effects [33].

Shin et al. [35] suggested early prediction of PTDM as the most effective and modifiable approach for managing PTDM, followed by rigorous glucose monitoring and management of a susceptible transplant population for PTDM. Their study used CGM to monitor the perioperative glycemic profile and dynamics and identify the risk factors for PTDM. They identified several baseline factors, such as age and sex, postoperative factors, and immunosensitive treatments influencing the risk of PTDM. Multivariate analysis also revealed that post-operative time above range (TAR; % time with glucose >10.0 mmol/L) was an independent risk factor for PTDM at 6 months post-kidney transplant (OR 1.17; 95% CI 1.06–1.29; *P* = 0.002) [35]. Additionally, preoperative indicators of hyperglycemia, including high glucose management indicator (GMI, a CGM-based calculated parameter approximately equivalent to HbA1c) and daily peak glucose levels, were significantly correlated with the risk of developing PTDM. CGM postoperatively outperformed conventional blood glucose monitoring in predicting PTDM [35].

Glucose changes in earlier weeks post-transplant have been explored as a risk factor for PTDM. A study using the conventional glucose measurement method revealed that fluctuating fasting plasma glucose variability in the first two weeks post-transplant is an independent risk factor for PTDM in kidney transplant recipients [36]. The value of CGM as an early predictive tool for the risk of developing diabetes or impaired glucose intolerance has been studied in 46 kidney transplant patients without pre-existing diabetes [37]. CGM metrics, particularly %TAR, were found to have better predictive value for PTDM and impaired glucose tolerance than HbA1c and fasting plasma glucose, particularly in the first seven days after transplant [37]. A TAR of 31.8% on day eight post-transplant yielded 88% sensitivity and 83% specificity for PTDM prediction, whereas a modified TAR with a lower threshold (glucose >7.8 mmol/L) and a value of 13.2% on day 30 provided 94% sensitivity but only 78% specificity for PTDM diagnosis [37].

Wojtusciszyn et al. [38] further explored the associations between early hyperglycemic changes after kidney transplantation and the risk of developing NODAT, using both CGM and capillary blood glucose measurements. The results showed that time with glucose >7.7 mmol/L measured by CGM in the first four days following transplantation did not correlate with the risk of NODAT within three months of kidney transplantation. However, CGM helped to identify patients who developed an impaired fasting glucose test or NODAT within three months post-transplant. Table 2 outlines studies assessing the predictive value of CGM for PTDM.

**Table 2:** Predictive value of continuous glucose monitoring (CGM) for post-transplant diabetes mellitus (PTDM)

Study	Year	Sample Size	CGM Timing	Metric(s) Used	Predictive Findings
Shin et al.	2024	60 were participants finally included (with no prior diagnoses of DM)	Peri- and post-op ( 14 days pre and 14 post-transplant)	TAR, GMI, peak glucose	Post-op TAR was independently predictive of PTDM at 6 months (OR 1.17, *p* = 0.002)
Eleftheriadis et al.	2024	46 participants were non-diabetic at time of transplantation	For average of 90 days post-transplant	%TAR	TAR at Day 8 (31.8%) predicted PTDM with 88% sensitivity, 83% specificity
Wojtusciszyn et al.	2013	43 non-diabetic KT recipients	First 4 days post-KT	Mean glucose & TAR %	Non predictive methodology, higher mean glucose tended to higher in review who developed NODAT at 3 months post-transplant

Shin et al. [35] presented a well-designed study with a standardized population at different levels and a strong predictive methodology that considered multiple relevant factors. However, the study did not consider dietary factors, carbohydrate consumption, or the participants’ physical activity levels, and the CGM data were not blinded, potentially affecting glucose measurements. Eleftheriadis et al. [37] conducted extensive CGM monitoring in a reasonably sized heterogeneous cohort of 46 patients, using a robust methodology but without reference to dietary factors or carbohydrate intake. Although their work demonstrated satisfactory diagnostic performance, no multivariable modelling was performed, and the analysis focused more on diagnostic accuracy than on true predictions. Additionally, the absence of stratification based on preoperative HbA1c could potentially limit the accuracy of PTDM prediction.

## Long-term management and monitoring of blood glucose in kidney transplant recipients

In one randomized clinical trial, CGM was used as a treatment-guiding tool, it demonstrated that divided prednisolone dosing (twice-daily regime of the total requirements) significantly reduces glycemic variability and hyperglycemia compared with once-daily dosing. The participants were monitored for five days post-kidney transplant [39]. This trial highlights the potential of CGM as a valuable tool for future studies that involve the modification of medication-related risk factors for PTDM and for assessing individualized immunosuppressive strategies.

The Kidney Disease Improving Global Outcomes (KDIGO) guidelines recommend screening for NODAT in kidney transplant recipients, using fasting blood glucose, oral glucose tolerance tests, and/or HbA1c measurements weekly in the first month post-transplant, every three months in the first year, and at least annually thereafter. It is also recommended that screening occurs whenever there is a substantial increase in the dose of calcineurin inhibitors, mTOR inhibitors, or corticosteroids [32].

The 2014 International Consensus Guidelines suggest regular fasting or pre-meal glucose testing in the first three months in patients with early hyperglycemia and that HbA1c measurement performs poorly in the first three months due to factors such as anemia, kidney graft function, and latency. It can be used after three months, initially every month until one year post-kidney transplantation, and then annually. The guidelines recommend closer monitoring with lifestyle modification if HbA1c falls within the prediabetic range [18].

The guidelines of the Association of British Clinical Diabetologists (ABCD) and Renal Association for the detection of PTDM after kidney transplant align with the recommendations of the American Diabetes Association (ADA) and the International Consensus Guidelines [19]. The ABCD/RA guidelines do not currently include CGM as a recommended tool for the detection and management of PTDM, although the guidance acknowledges the potential role of CGM in detecting early hyperglycemia and glycemic variability, especially when other measurements may be unreliable.

The use of CGM in kidney transplant recipients to identify hyperglycemic events and to aid early treatment that would have gone unnoticed by standard laboratory testing was reported in two investigations, one involving children and the other involving adults [26, 40]. Pasti et al. [40] emphasized the need for further research into the potential role of CGM in reducing the risk of cardiovascular complications and improving patient and graft survival.

The Association of British Clinical Diabetologists (ABCD) and the Renal Association’s guidelines advise against diagnosing PTDM early in the post-transplant period due to the common occurrence of transient hyperglycemia in the first few weeks; therefore, diagnosis is typically delayed until six weeks post-transplant. This aligns with the American Diabetes Association’s recommendation to formally diagnose PTDM once the patient is stable on immunosuppressants and in the absence of stress [41]. While CGM is not intended as a diagnostic tool for PTDM, it has demonstrated value in detecting glycemic variability and hyperglycemic episodes that might otherwise go unnoticed by traditional methods [19].

Articles also addressed CGM in recipients with pre-diabetes and those who had diabetes before the transplant. A study reported that CGM use in transplant recipients with pre-existing diabetes was associated with improved HbA1c and GMI. This highlights the potential of CGM to improve glycemic profile outcomes in the pre-diabetic population. This study emphasized the need for further research on the impact of CGM on mortality and graft survival in kidney transplant patients [42].

A randomized clinical trial was conducted to assess glycemic control in the first days after kidney transplant, comparing CGM with traditional finger-stick glucose monitoring in post-renal transplant patients with diabetes. The CGM group presented significantly lower daily mean glucose levels and fewer hyperglycemic episodes in the first few days post-transplant, although there were no significant differences in hypoglycemia, infections, or length of hospital stay post-kidney transplant. These findings support CGM as a more precise tool in glucose management post kidney transplantation [43]. The comparative findings between the CGM and standard methods are shown in Table 3.

**Table 3:** Comparison of CGM and conventional glucose monitoring methods in kidney transplant recipients

Study	Year	Comparator	Population	CGM Advantage	Notes
Rodríguez et al.	2010	Standard glucose checks	SPK vs. KT	Detected more hyperglycemia and GV	SPK had fewer glucose excursions
Werzowa et al.	2012	HbA1c, FPG	KT with diabetes	CGM revealed more hyper events	HbA1c underreported high glucose
Jandovitz et al.	2023	Fingerstick glucose	Post-KT diabetics	Lower daily glucose & fewer peaks	RCT; no difference in hypoglycemia or LOS
Clayton et al.	2014	OGTT, HbA1c	First 3 months post-KT	Detected early hyperglycemia missed by others	OGTT/HbA1c misclassified patients
Agate et al.	2022	HbA1c vs. CGM (GMI)	Preexisting diabetes post-KT	Improved control with CGM	Need for CGM-personalized care plans

**Table 4:** Glycemic profile monitoring in transplant recipients using continuous glucose monitoring (CGM)

Study	Year	Population	Comparator	CGM Findings	Interpretation
Yang et al.	2019	KT vs. LT (non-diabetics)	None	KT had higher early mean glucose levels	KT patients more prone to early diabetes
Jin et al.	2019	KT vs. LT	None	Greater glucose excursions & MAG in KT	CGM showed KT had more glycemic instability
Werzowa et al.	2012	Diabetic KT recipients	FPG, HbA1c	More hyperglycemic episodes and higher AUC detected	HbA1c poorly reflected real glucose burden
Clayton et al.	2014	KT recipients (first 3 months)	OGTT, HbA1c	CGM revealed early hyperglycemia; OGTT/HbA1c missed cases	CGM better for early monitoring post-KT

Although, to our knowledge, no studies have directly assessed the burden of CGM use in kidney transplant recipients, evidence from the diabetes population has identified several practical and psychological challenges for CGM, such as device handling issues, skin irritation, and anxiety related to continuous glucose data [44]. Given the massive lifestyle and complex medication changes during the immediate post-transplant period, it is reasonable to assume that similar CGM-related factors may contribute to the treatment burden in this group.

CGM represents a new advanced technology in diabetes management; however, it is equally important to consider the challenges associated with its implementation in different populations. A recently published systematic review addressing the barriers to CGM use among individuals with diabetes identified several barriers, with the financial cost being one of the most significant. The review highlighted that the majority of the available literature originates from developed countries, whereas the literature from developing countries remains notably limited. Financial barriers occur at multiple levels, including the cost of sensors and supporting technology as well as the absence of governmental or insurance support [44].

In addition to the cost of the technology, the review identified several individual and organizational factors that may hinder CGM uptake. These include fear of sensor insertion, inadequate technical and social support, limited training and knowledge in interpreting CGM data, disruptive alarms, reduced accessibility for users with visual impairments, and limited access to required technologies such as smartphones [44].

More research and trials are needed, especially to assess the effectiveness of CGM-guided individualized immunotherapy on a wider scale. An effectiveness study in controlling hyperglycemic kidney transplant populations must take into account and employ a prospective study design that incorporates randomized data. Establishing cost-effectiveness data related to kidney transplant recipients and developing transplant-specific CGM education frameworks will be essential to translate these promising findings into routine care pathways.

## Glycemic profile monitoring in transplant recipients: findings from continuous glucose monitoring (CGM)

The following literature highlights the utility of CGM metrics and approaches for studying the glucose profile of transplant recipients through comparisons with traditional glucose measurements such as HbA1c and fasting plasma glucose (FPG). One study compared the early post-transplantation onset of diabetes and glycemic profile in patients without pre-onset diabetes or pre-diabetes who underwent kidney (KT) and liver transplants (LT). The incidence of early new-onset diabetes was higher in the KT group than in the LT group, at 42.1% and 16.7%, respectively. The average mean glucose level was significantly higher in the KT than in LT recipients in the early period post-transplant [45]. Similar findings have been reported, indicating that the incidence of high mean glucose excursion and mean absolute glucose using CGM post-solid organ transplant organs was significantly higher in kidney transplant recipients than in liver transplant recipients [46]. Another study reported that CGM identified significantly more hyperglycemic episodes, higher glucose levels, and a greater area under the curve (AUC) for glucose in diabetic renal transplant patients than did FPG and HbA1c. HbA1c testing following renal transplantation is poorly correlated with CGM data and often misclassifies patients as normal [47].

An analysis further explored the glycemic profile by comparing traditional tests to CGM monitoring in the first three months after kidney transplantation. The study revealed that HbA1c measurements taken at three months post-transplant were poorly correlated with CGM glucose in the first month post-transplantation. CGM showed that hyperglycemia is common in the first three months post-transplant, and a normal three-month OGTT could not reliably rule out hyperglycemia [48]. Table (4) summarizes CGM findings on glycemic profiles post-transplant.

Comparative analyses consistently revealed that CGM identified a greater burden of hyperglycemia and variability than did HbA1c or fasting glucose testing, particularly within the first three months post-transplant. These discrepancies confirm that reliance on traditional assays may underestimate early metabolic risk. Although the study designs remain small and short-term, the reproducible pattern of poor correlation between CGM and HbA1c supports the notion that CGM captures clinically relevant fluctuations invisible to standard metrics. Future investigations should focus on harmonizing CGM endpoints and establishing consensus definitions for glycemic targets specific to the transplant population.

### Limitations of the review

As a narrative synthesis, it did not follow a systematic review synthesis, and no formal risk-of- bias assessment was performed. The current evidence regarding CGM use in kidney transplant recipients is limited by small sample sizes, heterogeneity of study designs, and the variety of CGM metrics and patient populations. Most of the studies are observational and short-term and lack key confounder adjustment. Few randomized trials have been conducted, and publication bias may exist.

## Conclusion

CGM offers advantages over other methods of glucose monitoring in post-transplant patients owing to the unreliability of HbA1c in early weeks. It aids glucose monitoring and management in the kidney transplant population, particularly those with PTDM or preexisting diabetes. CGM real-time monitoring of glycemic trends allows for early intervention, which could benefit the reduction of complications such as graft rejection, cardiovascular events, and chronic hyperglycemia-related damage. The CGM provides potential predictive insights into the development of PTDM.

Given that CGM appears promising for enhancing the glycemic management of kidney transplant recipients, additional research is needed to explore its clinical implications in practice. Large, multicenter, randomized controlled trials are needed to assess whether CGM-guided post-transplant management can improve graft survival mortality and enhance cardiovascular outcomes. Future studies should also evaluate the cost-effectiveness of routine CGM use in the peri-transplant and outpatient settings, as economic considerations may influence adoption in clinical practice. Furthermore, research should investigate the incorporation of CGM metrics into clinical decision-making, modifications to immunosuppressive therapy, dietary practices, physical activity, and glucose-lowering pharmacotherapy. More robust methodological approaches are required to assess the ability of CGM to predict PTDM and to determine whether early prediction can support preventive interventions, such as earlier initiation of insulin or glucose-lowering therapies.

Based on current evidence, CGM may be used as a complementary monitoring tool in renal transplant programs. Early postoperative use can help detect unrecognized hyperglycemia and may assist in assessing glycemic variability and identifying PTDM risk. However, given the limitations of existing studies, CGM should be integrated alongside standard monitoring, and further research is needed to determine its definitive clinical impact and cost-effectiveness for kidney transplant recipients.

## Data Availability

No datasets were generated or analysed during the current study.

## Abbreviations

ABCD: Association of British Clinical Diabetologists
ADA: American Diabetes Association
CGM: Continuous glucose monitoring
ESRD: End stage renal disease
FPG: Fasting plasma glucose
GMI: Glucose management indicator
GV: Glycemic variability
HbA1c: Hemoglobin A1c
KDIGO: Kidney Disease: Improving Global Outcomes
mTOR: Mammalian target of rapamycin
NODAT: New-onset diabetes after transplant
OGTT: Oral glucose tolerance test
PTDM: Post-transplant diabetes mellitus
RA: Renal association
SPK: Simultaneous pancreas – kidney transplantation
TAR: Time above range
T2DM: Type 2 diabetes mellitus
MAGE: Mean amplitude of glycemic excursions
